# Change in Glycemic Control With Use of a Digital Therapeutic in Adults With Type 2 Diabetes: Cohort Study

**DOI:** 10.2196/diabetes.9591

**Published:** 2018-02-14

**Authors:** Mark A Berman, Nicole L Guthrie, Katherine L Edwards, Kevin J Appelbaum, Valentine Y Njike, David M Eisenberg, David L Katz

**Affiliations:** 1 Better Therapeutics LLC San Francisco, CA United States; 2 Prevention Research Center Griffin Hospital Yale University Derby, CT United States; 3 Department of Nutrition Harvard TH Chan School of Public Health Harvard University Boston, MA United States

**Keywords:** type 2 diabetes, mobile health, mHealth, lifestyle medicine, mobile apps, digital therapeutics

## Abstract

**Background:**

Intensive lifestyle change can treat and even reverse type 2 diabetes. Digital therapeutics have the potential to deliver lifestyle as medicine for diabetes at scale.

**Objective:**

This 12-week study investigates the effects of a novel digital therapeutic, FareWell, on hemoglobin A_1c_ (HbA_1c_) and diabetes medication use.

**Methods:**

Adults with type 2 diabetes and a mobile phone were recruited throughout the United States using Facebook advertisements. The intervention aim was to effect a sustainable shift to a plant-based dietary pattern and regular exercise by advancing culinary literacy and lifestyle skill acquisition. The intervention was delivered by an app paired with specialized human support, also delivered digitally. Health coaching was provided every 2 weeks by telephone, and a clinical team was available for participants requiring additional support. Participants self-reported current medications and HbA_1c_ at the beginning and end of the 12-week program. Self-efficacy related to managing diabetes and maintaining dietary changes was assessed via survey. Engagement was recorded automatically through the app.

**Results:**

We enrolled 118 participants with a baseline HbA_1c_ >6.5%. Participants were 81.4% female (96/118) and resided in 38 US states with a mean age of 50.7 (SD 9.4) years, baseline body mass index of 38.1 (SD 8.8) kg/m^2^, and baseline HbA_1c_ of 8.1% (SD 1.6). At 12 weeks, 86.2% (94/109) of participants were still using the app. Mean change in HbA_1c_ was –0.8% (97/101, SD 1.3, *P*<.001) for those reporting end-study data. For participants with a baseline HbA_1c_ >7.0% who did not change medications midstudy, HbA_1c_ change was –1.1% (67/69, SD 1.4, *P*<.001). The proportion of participants with an end-study HbA_1c_ <6.5% was 28% (22/97). After completion of the intervention, 17% (16/97) of participants reported a decrease in diabetic medication while 8% (8/97) reported an increase. A total of 57% (55/97) of participants achieved a composite outcome of reducing HbA_1c_, reducing diabetic medication use, or both; 92% (90/98) reported greater confidence in their ability to manage their diabetes compared to before the program, and 91% (89/98) reported greater confidence in their ability to maintain a healthy dietary pattern. Participants engaged with the app an average of 4.3 times per day. We observed a significantly greater decrease in HbA_1c_ among participants in the highest tertile of app engagement compared to those in the lowest tertile of app engagement (*P*=.03).

**Conclusions:**

Clinically meaningful reductions in HbA_1c_ were observed with use of the FareWell digital therapeutic. Greater glycemic control was observed with increasing app engagement. Engagement and retention were both high in this widely distributed sample.

## Introduction

Type 2 diabetes prevalence is at pandemic levels and continues to rise here in the United States and globally [1,2]. Medication costs are rising in parallel and threaten to bankrupt national health systems [3,4]. Despite increased use of medications and the advent of new pharmacological treatments, glycemic control among those with diabetes does not appear to be improving since 2010 [5].

While type 2 diabetes is currently considered a chronic progressive disease that typically requires increasing medications over time [6], there is also growing evidence that type 2 diabetes is treatable, and in some cases reversible, with comprehensive lifestyle changes alone [7-15]. Therapeutic lifestyle changes include substantial improvements in dietary pattern, activity, and exercise; avoidance of tobacco and excess alcohol; and additional behaviors that improve sleep, stress, mood, and social connection [9,13,16].

The practice of leveraging therapeutic lifestyle changes as medicine is often referred to as lifestyle medicine. The case for lifestyle as medicine has been detailed elsewhere and applies not just to type 2 diabetes but to many other lifestyle-related chronic diseases, which collectively account for roughly 80% of premature mortality and health care costs [16-19].

An intervention that successfully delivers lifestyle therapy has potential benefits over traditional therapeutics like medications and surgery. Potential benefits include a more favorable side-effect profile due to fewer adverse effects and additional non–disease-specific health benefits, lowered health care costs, and for many, greater acceptability [16-19].

Lifestyle therapy has been shown to outperform pharmacotherapy in diabetes prevention [20,21] although the challenge of translating that result to real-world populations persists [22,23]. For diabetes reversal, there is similar opportunity but less clarity about the preferred approach [10,15], and thus there are few widely accessible, cost-effective therapies available [16]. Digital therapeutics that deliver lifestyle therapy have potential to fill this therapeutic void because they are inherently scalable therapies that can be accessed outside of traditional brick-and-mortar constraints (ie, wherever a patient goes, at any moment in time).

A digital therapeutic has been described as an intervention for treating disease that is delivered continuously through digital means [24,25]. This study examines a digital therapeutic, called FareWell, that aims to effect a sustainable shift to a whole food, plant-based dietary pattern and regular exercise by advancing culinary literacy and lifestyle skill acquisition. It incorporates interactive mobile computing (ie, an app), remote sensors (eg, wearable devices and home monitors), and human care (eg, health coaching) delivered by digital means. This solution affords for population management and specialized care that can be made accessible to adults living in a vast geography, at scale. As envisioned, it is intended as a stand-alone intervention that could replace or complement other interventions.

In this study, we sought to understand to what degree a novel, skill-focused, digital therapeutic could change HbA_1c_ and antidiabetic medication use in a geographically widely distributed sample of adults with type 2 diabetes. While the ultimate goal of the intervention is to be more cost effective than other interventions, this study examines effectiveness alone.

## Methods

### Trial Design and Participants

We conducted a 12-week, nonblinded, single-arm interventional study in a convenience sample of adults with a self-reported diagnosis of type 2 diabetes.

Participants were recruited online through advertisements listed on Facebook and to a lesser extent Craigslist, targeted to adults in any US state with an interest in type 2 diabetes. The study was described as evaluating a free 3-month lifestyle change program that uses digital tools, a plant-based dietary pattern, and health coaching.

Eligibility criteria included having a diagnosis of type 2 diabetes, age 18 years or older, and possession of an Android or iPhone mobile phone as demonstrated by the ability to download the intervention app. Type 2 diabetes status was presumed by the combination of a self-reported diagnosis and an initial HbA_1c_ of 6.5% or higher. Participants were excluded if they were not able to comply with the study protocol—for example, if they could not speak or read English or did not have sufficient computer literacy to operate the app successfully.

Enrollment was on a first-come-first-served basis and all data collection occurred online via electronic survey or directly through the app. Participants who were interested in the study were invited to download the app and enter a code to unlock the app. Participants were then instructed by the app to create an account using their email address. Upon creating an account, participants were emailed an informed consent document to review. Informed consent was obtained for each study participant via discussion with a study staff member prior to commencing their first coaching call. This phone call with study staff also ensured that each participant was unique.

An incentive of US $200 was offered to participants who participated in the program and completed data reporting at 3 months. The study was approved and overseen by Quorum Review Institutional Review Board [26], an independent ethics review board located in Seattle, Washington.

### Intervention App Development

The intervention app was developed by a San Francisco–based startup of which the authors are founders and/or employees or scientific consultants. The first version of the app was developed as a Web app using responsive design and validated with usability testing, followed by a pilot clinical trial in adults with class 1 obesity and elevated risk for metabolic disease [27]. It was then redeveloped as a native app for Android and iOS using human-centered software design principles [28] and subject to basic usability testing prior to the start of this study.

Periodic updates of the app were released during the study period. Study participants enrolled using version 1.3 of the app and completed the study on version 1.5. The vast majority of the changes in the app during the study period were minor experience improvements or bug fixes. One new feature—an artificially intelligent conversational bot—was released in the last month of the study in v1.5 along with the ability to enter home finger-stick readings. This bot enabled a new method for participants to report meals eaten and visualize the number of healthy meals eaten each week.

### Intervention

The digital therapeutic consists of use of the intervention app paired with specialized human support, also delivered digitally. The content design of both app and human support incorporated evidenced-based dietary and lifestyle recommendations such as a dietary pattern consisting mainly of whole food plant-based meals and regular exercise meeting or exceeding national guidelines [9,13,19]. Since it is known that increased meals prepared at home is associated with decreased disease burden [29], additional content was developed with expert input to enhance culinary skill acquisition with the aim of increasing meals prepared at home.

Several theoretical models informed the design of app features, including the theory of planned behavior (eg, features were designed to alter intentions), social cognitive theory (eg, features were designed to enhance self-efficacy, enable experiential learning, and reinforce healthy behaviors), and behavioral economics (eg, use of default choices). Both the app and accompanying human support are designed as a learning platform, which aims to impart the lifestyle skills necessary to reverse cardiometabolic disease.

The app was designed primarily to facilitate the learning and adoption of plant-based meals, self-monitoring habits, and scheduling of coaching calls. It is intended to be used ad libitum, but expectations of use were established during the informed consent process as follows:

Use of the meal planning feature that facilitates advanced planning of meals and automated shopping lists (approximately 5 minutes per week). The meal planning feature uses default recipes that met prespecified criteria for ease-of-preparation, inclusion of easy-to-access, whole food, plant-based ingredients, and staged introduction of culinary techniques. Participants could easily swap meals or plan to eat a meal not in the recipe database. An interactive shopping list was autopopulated whenever a meal plan was created or modified.

Self-monitoring of weight daily (via digitally connected scale provided free to participants or by self-report in app) and the option of reporting meals made (approximately 1 to 2 minutes per day).

Reviewing of educational materials aimed at advancing culinary or health literacy (approximately 15 to 20 minutes per week).

An optional, private Facebook community was created to provide additional peer-to-peer and expert-to-peer support (ad libitum).

The app delivered reminders—for example, to schedule a coaching call or report meals made or eaten—in the form of in-app notifications and an ability to message the participant’s health coach.

The primary form of human support was delivered by 30-minute telephonic health coaching calls, scheduled at the participant’s convenience every 2 weeks via the study app. Health coaching is an evidence-based practice grounded in behavior change theory that uses guided conversational techniques such as motivational interviewing [30,31]. All study health coaches had completed training from accredited health coaching institutions and received additional training in lifestyle and culinary medicine, research methods, and training for coaching within a clinical team prior to the start of the study.

Health coaching calls were used to set and review personalized behavioral goals with each participant. These goals centered largely on the attainment of dietary skills and repetition for habit formation but also included setting physical activity goals and addressing barriers to these goals. For example, participants worked with their coach to establish an individualized plan to progressively reach or exceed a goal of 30 minutes of moderately intense physical activity per day.

During the intervention period, the health coaches were supported by a specialized team of lifestyle medicine experts including a nurse practitioner, internist, psychiatrist, chef-educator, and registered dietitian who were also available to speak to members on an as-needed basis via a care-escalation process. Participants were asked to continue managing all medications with their primary care team or endocrinologist during the course of the study.

## Measures

### Demographics

Participants reported age, gender, height, weight, and US state of residence as a part of the sign-up process for the study app.

### Hemoglobin A_1c_ and Medication Use

Most recent HbA_1c_ and current diabetic medication use (name, dose, and frequency of medication) were self-reported in the study app by participants. Participants were encouraged by their coaches to report any changes to medications within their study app. In addition to in-app coach messages, email reminders were used to prompt entry of a follow-up HbA_1c_ and updated medications at 12 weeks. Medication and HbA_1c_ data were reviewed by 2 study authors (NLG, MAB). Participants were contacted by study staff (KLE, NLG) to help clarify potential reporting errors.

### Engagement

Engagement with both the study app and coaching calls was measured automatically via the study app. Total engagement is defined as the average number of recorded app actions per day (eg, planning or reporting meals, scheduling calls, building shopping lists).

### Satisfaction

All participants were invited to fill out a Net Promoter Score (NPS) survey [32] at week 10 after sign-up. The NPS consists of 1 question “How likely are you to recommend FareWell to a friend?” rated on a 10-point scale (0-6=detractors, 7-8=passives, 9-10=promoters). The NPS is calculated by subtracting the percentage of detractors from the percentage of promoters.

### Self-Efficacy

End of program self-efficacy to manage diabetes and maintain an optimal dietary pattern was measured via online survey questions using a Likert scale; survey was emailed to participants during their 12th week.

### Statistical Methods

Statistical analyses were performed using SAS software version 9.4 (SAS Institute Inc). Change over time of continuous variables was analyzed using 2-tailed paired Student *t* tests with alpha set at .05 and chi-square tests for differences in categorical variables. The McNemar test was used to evaluate medication change.

To evaluate the combined effects of medication and HbA_1c_ change, we calculated a composite outcome measure defined as a decrease in diabetic medication use without an increase in HbA_1c_ or an improvement in HbA_1c_ of at least 0.5% without an increase in diabetic medication use.

We used mixed-effects modeling to test the effects of baseline body mass index (BMI), years since diagnosis of diabetes, net change in diabetes medications, total app engagement, and baseline HbA_1c_ on the mean change in HbA_1c_. To evaluate the intent-to-treat effect, we used a last-value-carried-forward approach for the missing data from participants who did not report follow-up HbA_1c_ levels. Since effect-size can be modulated by baseline HbA_1c_ [33], we also tested the effects of a log transformed HbA_1c_.

To investigate the relationship between engagement with the program and HbA_1c_, we first defined tertiles of app engagement using the sum of all actions taken in the app during the study. A general linear regression was used to test the effect of app use tertile with the change in HbA_1c_. Change in HbA_1c_ was set as the dependent variable with tertile of app engagement and the log transformed baseline HbA_1c_ as independent variables. Using the least square means pairwise comparison, we tested the differences in changes in HbA_1c_ by the tertiles of app engagement.

## Results

### Participants

A total of 123 individuals with self-reported type 2 diabetes and an initial HbA_1c_ of 6.5% or higher downloaded the intervention app, of which 118 (95.9% of downloads) consented to participation in the study. Of the consented participants, 113 were recruited from Facebook and 5 from Craigslist. There were 9 dropouts (7.6% of consented) during the study. Reasons for dropping out were participant not feeling ready to make lifestyle changes (5), difficulty using the app (2), and no reason given (2). Of the remaining 109 participants, 94 (86.2%) were still using the app at 12 weeks, and 101 (92.7%) provided some or all end-study data.

There were no adverse events observed thought to be related to the study intervention. However, 2 adverse events were reported during the first month of study period. One participant reported suicidal ideations to a coach, and another participant was hospitalized briefly for dehydration after a flu-like illness. Both participants recovered fully from their events and were able to continue participating in the study.

Baseline characteristics are summarized in Table 1. Participants from 38 US states consented to participate; 81.4% (96/118) were female, with a mean age of 50.7 (SD 9.4) years, mean BMI of 38.1 (SD 8.8) kg/m^2^, and mean HbA_1c_ of 8.1% (SD 1.6) at baseline. There were no statistical differences in baseline characteristics between those who consented and those who submitted end-study data.

**Table 1:** Sample characteristics at baseline by program completion.

User characteristics	Total n=118	Completed program n=109	Submitted end-study data^a^ n=101	*P* value^b^
Female, n (%)	96 (81.4)	87 (79.8)	80 (79.2)	.14
Age (years), mean (SD)	50.7 (9.4)	50.4 (9.6)	50.4 (9.7)	.85
Geographic distribution, # US states	38	37	37	.71
Hemoglobin A_1c_ (%), mean (SD)	8.1 (1.6)	8.2 (1.6)	8.2 (1.7)	.81
Body mass index (kg/m^2^), mean (SD)	38.1 (8.8)	38.4 (9.0)	38.1 (8.9)	.99
Time since diabetes diagnosis (years), mean (SD)	2.6 (1.6)	2.6 (1.5)	2.6 (1.5)	.99
Diabetes medications (count), mean (SD)	1.4 (0.9)	1.5 (0.9)	1.5 (0.9)	.73

^a^Participants who submitted an end-study hemoglobin A_1c_ and/or self-efficacy survey.

^b^*P* value comparing total sample to those submitting end-study data.

### Hemoglobin A_1c_

Among participants who reported an end-study HbA_1c_, 80% (78/97) had improvement of HbA_1c_, with 59% (57/97) having a decrease of 0.5% or more, 39% (38/97) having a decrease of 1% or more, and 23% (22/97) having a follow-up HbA_1c_ <6.5%. The mean change was –0.8% (SD 1.3, *P*<.001) over a mean interval of 3.5 (SD 0.9) months. This change remained statistically significant in our mixed-effects model (*P*=.003). Substituting the log transformed baseline HbA_1c_, we found that the impact of baseline HbA_1c_ was modulated and the significance of the mean change in HbA_1c_ was improved (*P*<.001). Using a last-value-carried-forward approach for the missing data from participants who did not report follow-up HbA_1c_ levels, the mean change remained statistically significant (118/118, –0.6%, SD 0.9, *P*<.001).

Among those with a baseline HbA_1c_ >7%, the mean change was –1.0% (n=69, SD 1.4, *P*<.001). Excluding those who experienced a change in glycemic medication midstudy (2/69), the mean change in HbA_1c_ was –1.1% (67/69, SD 1.4, *P*<.001).

### Medication Use

At the start of the study, participants reported taking an average of 1.4 (SD 0.9) diabetic medications with a self-reported average time since diagnosis of 2.6 (SD 1.6) years. Of those reporting follow-up medication data, 4% (4/97) changed medications or dosages within the 12-week study (ie, their medication changes were likely to impact follow-up HbA_1c_). In conjunction with reporting an end-study HbA_1c_, 17% (16/97) of participants reported decreasing or stopping 1 or more diabetic medications and 8% (8/97) increased or added 1 or more diabetic medications. The frequency of decreased medication use (either decreasing dose or stopping a medication) compared to baseline medication use was statistically significant (*P*<.001).

Using the composite outcome measure defined above, 57% of participants (55/97) met the composite outcome of reducing HbA_1c_, reducing diabetic medication use, or both.

### Program Engagement and Satisfaction

Of the individuals who consented to participate, 92.4% (109/118) were active in the study at the end of the 12-week intervention period and 86.2% (94/109) were still using the app. Total distinct app engagements averaged 4.3 (SD 2.5) per day, and average number of coaching calls completed was 4.1 (SD 1.8) during the 12-week period.

**Figure 1:**
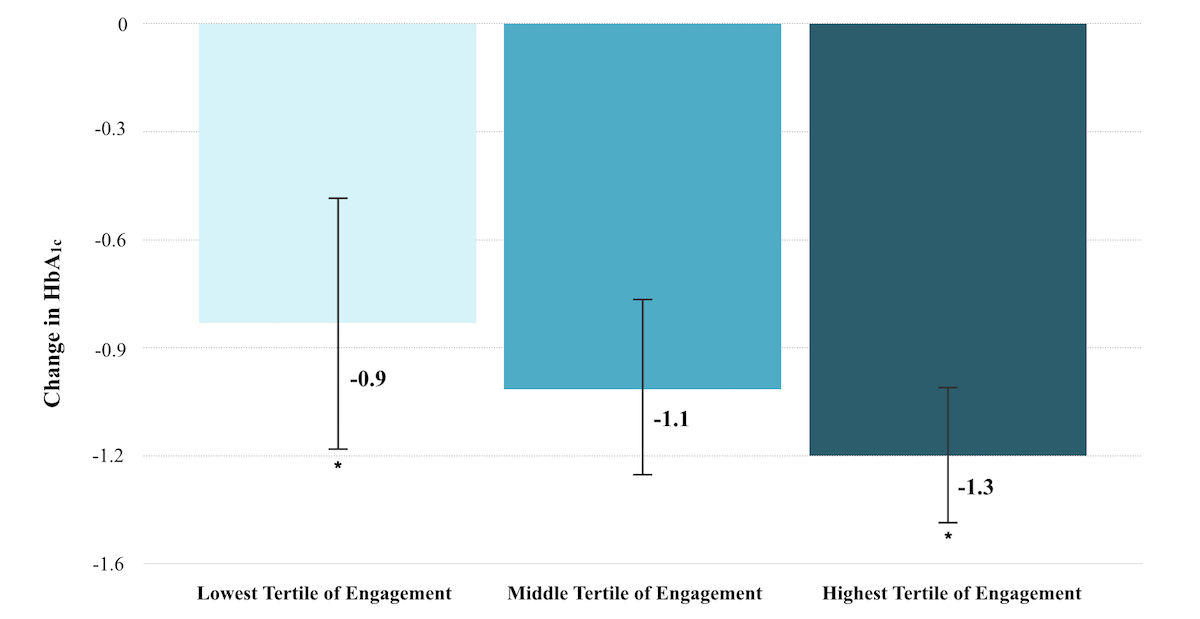
Change in hemoglobin A_1c_ by tertile of engagement in subset of participants with baseline HbA_1c_ >7.0% and no midstudy medication changes. Bars represent means and standard errors. Star indicates *P*=.03 between groups.

We explored the relationship between app use and HbA_1c_ change. There was a stepwise decrease in HbA_1c_ as app engagement level increased. For example, as displayed in Figure 1, in those with a baseline HbA_1c_ >7.0% who did not change medications during the study period, the lowest tertile of engagers reduced HbA_1c_ by 0.9% (SD 1.3), whereas the highest tertile of engagers reduced HbA_1c_ by 1.3% (SD 1.0, *P*=.03 using log transformed baseline HbA_1c_).

The NPS survey was completed by 47.7% (52/109) of participants with 82.7% (43/52) of respondents giving a promoter score (9 or 10), 11.5% (6/52) a neutral score (7 or 8), and 5.8% (3/52) a detractor score (6 or below). The calculated NPS was 76.9%.

**Table 2:** Changes in hemoglobin A_1c_, diabetes medications, and self-efficacy.

Measures		Value	n	*P* value^a^
**Hemoglobin A_1c_ (%), mean change (SD)**		–0.8 (1.3)	97	<.001
	Duration (months)^b^, mean (SD)	3.5 (0.8)	97	
	Decrease by 0.5% or more, %	58.8	57	
	Decrease by 1.0% or more, %	39.2	38	
Decrease in diabetes medication use^c^, %		16.5	16	<.001
Increase in diabetes medication use^c^, %		8.3	8	
Daily mobile app engagements^d^, mean (SD)		4.3 (2.5)	109	
Diabetes self-efficacy^e^, mean (SD)		4.5 (0.6)	98	
Dietary change self-efficacy^e^, mean (SD)		4.4 (0.8)	98	

^a^Comparison of baseline and end-study values by paired Student *t* test for HbA_1c_, by McNemar test for medication use.

^b^Time between the baseline and end-study HbA_1c_ values.

^c^Includes those who changed dose and/or number of medications used.

^d^Includes use of all features in the mobile app; does not count log-in.

^e^Rated on a 5-point Likert scale with 5=a lot more confident and 1=a lot less confident.

### Self-Efficacy

Of the participants answering questions pertaining to self-efficacy, 92% (90/98) of those responding reported greater confidence in their ability to manage their diabetes compared to before the program, and 91% (89/98) reported greater confidence in their ability to maintain a healthy dietary pattern. Table 2 summarizes change in HbA_1c_, diabetes medications, and self-efficacy.

## Discussion

### Principal Findings

In this study, we examined the effectiveness of a digital therapeutic delivered to participants with type 2 diabetes distributed across the United States. We found clinically meaningful reductions in both HbA_1c_ and the proportion of participants who reduced diabetic medication use at the conclusion of the 12-week study period. We also observed greater glycemic control in participants with higher levels of engagement with the app.

The magnitude of HbA_1c_ reduction observed was comparable to those found with commonly prescribed medications [33,34] and successful intensive lifestyle interventions delivered in person [10]. In addition, a meaningful percentage (28%, 22/97) of participants achieved an HbA_1c_ value below the diabetic range, 23% (5/22) of whom reported no diabetic medication use, indicating potential for partial or complete remission of diabetes as defined by the American Diabetes Association consensus definition [35]. However, the short duration of this trial and lack of knowledge of the temporal sequence of lab test versus medication change does not allow us to evaluate remission status.

While this study supports the findings of others [36,37] who have demonstrated the efficacy of digital health apps, this is the first digital therapeutic study to our knowledge that emphasized a skill-building process according to the principles of lifestyle medicine rather than calorie or macronutrient counting or restrictions, meal replacements, or mandatory finger-stick monitoring. This is important because many situations that are not conducive to long-term health can ameliorate glycemic measures in the short term, among them starvation and serious infectious disease [38]. Part of the novelty of this intervention was use of a lifestyle approach to treat and reverse diabetes in the short term that is known to be compatible with overall health [18,19] and diabetes prevention [20,21] in the long term.

### Strengths and Limitations

The main limitations of this study stem from its single sample, nonrandomized design, self-selection of participants, and reliance on self-reported biometrics. As such, this study cannot establish causation nor can it rule out all potential confounders. In addition, in this short duration study, we did not independently quantify exercise or calorie-nutrient profiles and therefore cannot comment on the precise mechanisms of action.

The strength of this study is a design that closely mirrors real-world implementation of the intervention. The same clinical team and processes used in the study are used in real-world implementation of this digital therapeutic. And just like in the real world, the app continued to develop and experience bugs and bug fixes during the course of the study. This pragmatic study design in concert with recruitment of participants in 38 US states suggests generalizable findings. Other strengths of this study include high rates of retention and successful data collection.

### Conclusions

Future research in the form of randomized controlled trials will be needed to establish comparative effectiveness. In addition, longer duration trials will be needed to assess the durability of the lifestyle, biometric, and medication changes observed among diverse socioeconomic populations. Equally important will be research evaluating cost effectiveness. Finally, because this study evaluated an early version of a rapidly evolving digital therapeutic, it will be important to understand to what degree feature enhancements and additions modify the outcomes observed in this study.

## Abbreviations

BMI: body mass index
HbA_1c_: hemoglobin A_1c_
NPS: Net Promoter Score
